# *Leishmania* chitinase facilitates colonization of sand fly vectors and enhances transmission to mice

**DOI:** 10.1111/j.1462-5822.2008.01132.x

**Published:** 2008-06

**Authors:** Matthew E Rogers, Martina Hajmová, Manju B Joshi, Jovana Sadlova, Dennis M Dwyer, Petr Volf, Paul A Bates

**Affiliations:** 1 Liverpool School of Tropical Medicine Pembroke Place, Liverpool L3 5QA, UK; 2 Department of Parasitology, Faculty of Science, Charles University in Prague Vinicna 7, 128 44 Prague 2, Czech Republic; 3 Cell Biology Section, Laboratory of Parasitic Diseases, Division of Intramural Research, NIAID, National Institutes of Health Bethesda, Maryland 20892-0425, USA

## Abstract

Chitinases of trypanosomatid parasites have been proposed to fulfil various roles in their blood-feeding arthropod vectors but so far none have been directly tested using a molecular approach. We characterized the ability of *Leishmania mexicana* episomally transfected with *LmexCht1* (the *L. mexicana* chitinase gene) to survive and grow within the permissive sand fly vector, *Lutzomyia longipalpis*. Compared with control plasmid transfectants, the overexpression of chitinase was found to increase the average number of parasites per sand fly and accelerate the escape of parasites from the peritrophic matrix-enclosed blood meal as revealed by earlier arrival at the stomodeal valve. Such flies also exhibited increased damage to the structure of the stomodeal valve, which may facilitate transmission by regurgitation. When exposed individually to BALB/c mice, those flies with chitinase-overexpressing parasites spent on average 2.4–2.5 times longer in contact with their host during feeding, compared with flies with control infections. Furthermore, the lesions that resulted from these single fly bite infections were both significantly larger and with higher final parasite burdens than controls. These data show that chitinase is a multifunctional virulence factor for *L. mexicana* which assists its survival in *Lu. longipalpis*. Specifically, this enzyme enables the parasites to colonize the anterior midgut of the sand fly more quickly, modify the sand fly stomodeal valve and affect its blood feeding, all of which combine to enhance transmission.

## Introduction

Parasitic protozoa of the genus *Leishmania* are the causative agents of the leishmaniases and are transmitted between mammalian hosts by female phlebotomine sand flies. *Leishmania* infection can range from mild self-healing skin lesions to fatal visceral infection depending on the species of parasite. Currently, it is estimated that 12 million people worldwide are affected and two million new cases arise each year (WHO/TDR, 2004). Therefore, in the light of a very limited range of drugs and no effective vaccines, a detailed understanding of all aspects of these parasites' biology is desirable to formulate new antiparasitic strategies.

*Leishmania* exist in two main morphological forms: as non-motile intracellular amastigotes in mammalian mononuclear phagocytes; and as flagellated motile promastigotes in the gut of their sand fly vectors. Infection of a sand fly is initiated when amastigotes are acquired with an infectious blood meal, which then transform to promastigotes in 24–48 h. These promastigotes undergo multiplication and a complex series of transformations that culminates in the generation of mammal-infective metacyclic promastigotes, development being completed in 1–2 weeks (Sacks and Perkins, 1984; reviewed in Sacks and Kamhawi, 2001; Bates and Rogers, 2004; Kamhawi, 2006).

In order to complete their life cycle in the vector the parasites face various challenges. Among these they have to overcome two physical barriers. The first of these is the peritrophic matrix (PM), a meshwork of proteins and chitin secreted by the midgut epithelium that encloses the blood meal and therefore the parasites, shortly after feeding. The PM is a semi-permeable barrier allowing the inward diffusion of sand fly hydrolytic enzymes and outward diffusion of nutrients (Lehane, 1997), but prevents the egress of promastigotes. However, during the early phase of *Leishmania* development within the blood meal in the sand fly the intact PM is of benefit to parasite survival (Pimenta *et al*., 1997).The PM limits the rate of digestive enzyme diffusion and contact with parasites as they transform from amastigotes to promastigotes in the first 24–48 h, a brief phase during which they are vulnerable to proteolytic attack. Nevertheless, later in the infection an intact PM can act as a barrier to colonization of the anterior midgut, and must be negotiated before defecation of the digested blood meal. Although the PM will disintegrate eventually, this process was described to occur more quickly in infected sand flies (Walters *et al*., 1989; Schlein *et al*., 1991), indicating that the parasites may assist their own escape in some way. Conversely, in unnatural sand fly/*Leishmania* combinations where the PM did not break down before defecation, the infections were completely lost from these flies (Feng, 1951; Walters, 1993).

**Fig. 1:**
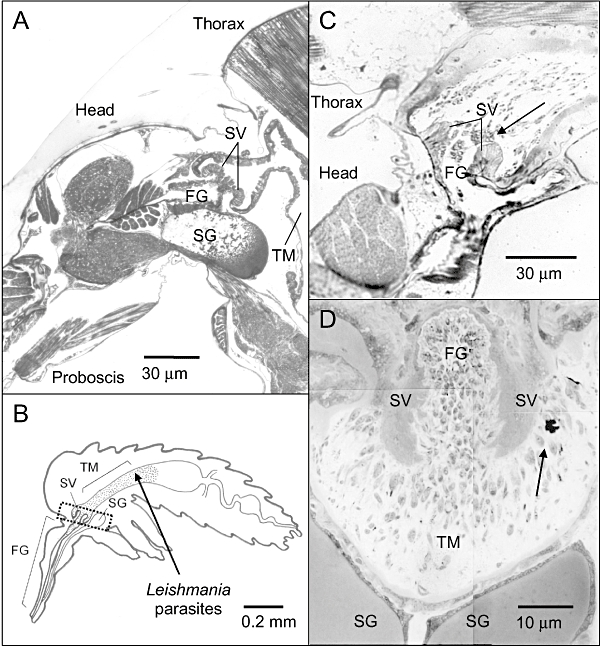
Anatomy of female sand flies. A. Light micrograph (×500 magnification) of longitudinal section through the head and thorax of an uninfected female sand fly showing the position of the stomodeal valve (SV) in relation to the salivary glands (SG), thoracic midgut (TM) and foregut (FG). B. Schematic of longitudinal section through a female sand fly highlighting the position occupied by a 7-day-old infection (stippled area). Dashed box highlights tissues of sand fly represented in (C) and (D). C and D. Sagital section (C) and composite cross-section image of light micrographs (D) (C: ×500; D: ×1000 magnification) from a mature wild-type *L. mexicana* infection showing the distension of the valve during infection. (D) was taken at an oblique angle through the infected oesophagus (foregut), stomodeal valve and thoracic midgut. Arrows point to examples of *Leishmania* promastigotes attached to and in the lumen of the sand fly gut. The salivary glands can be seen lying below the stomodeal valve and thoracic midgut.

The second physical barrier to completion of the life cycle is the stomodeal valve (SV), a cuticle-lined chitinous structure (Fig. 1). In sand flies the SV separates the midgut from the foregut and the proboscis. The valve is normally closed, only briefly opening to allow the inward passage of blood or sugar meals into the midgut during feeding. However, in infected sand flies the SV is colonized by various kinds of promastigotes, haptomonad promastigotes attaching to the chitinous surfaces of the valve and leptomonad/short-nectomonad promastigotes multiplying in the lumen of the anterior midgut (Molyneux and Killick-Kendrick, 1987; Gossage *et al*., 2003; Fig. 1). Further, in mature *Leishmania* infections the SV is forced open and becomes blocked with parasites embedded in promastigote secretory gel (PSG), a viscous mixture of phosphoglycans secreted by the parasites (Stierhof *et al*., 1999; Rogers *et al*., 2002; 2004). This opening of the SV is essential for colonization of the foregut and transmission by regurgitation (Rogers *et al*., 2004). Further, the SV appears to become physically damaged in infected sand flies (Schlein *et al*., 1992; Volf *et al*., 2004). As with the PM this damage has been proposed to promote transmission of the infection.

The PM and SV both include chitin as a major structural component. Therefore, the discovery of chitinase activity in *Leishmania* promastigote culture supernatants (Schlein *et al*., 1991; Shakarian and Dwyer, 1998; 2000) was of interest, and may be of relevance in aiding the parasites to overcome both of these barriers. Thus it was proposed that chitinase could locally digest the PM and assist the escape of promastigotes into the midgut lumen (Schlein *et al*., 1991). This was consistent with the effects of adding allosamidin, a chitinase-specific inhibitor, to the infectious blood meal of *Phlebotomus papatasi* (Pimenta *et al*., 1997). This had the effect of thickening the PM in uninfected flies, and prevented *Leishmania major* escaping from the blood meal in infected flies. However, as described above an interesting protective effect of the PM was also revealed in this study. Early parasite mortality within the blood meal was described and attributed to contact with sand fly midgut trypsins, an effect that was exacerbated by a reduction in the integrity of the chitin component of the PM (Pimenta *et al*., 1997). Conversely, allosamidin actually promoted early survival of parasites, even though they were later less able to escape the PM. Therefore, the chitin component of the PM appears to exert both a protective function and acts as a barrier to parasite development. However, the interpretation of this earlier work is complicated by the existence of sand fly chitinase (Ramalho-Ortigão *et al*., 2005), which could also contribute to some of the effects described.

The parasite chitinase has also been proposed to be responsible for damage to the SV (Schlein *et al*., 1992). In electron micrographs the electron-dense chitin layer of the SV shows evidence of degradation during *Leishmania* infection, suggesting that the damage is caused at least partially by the action of chitinolytic enzymes (Volf *et al*., 2004). However, despite these various proposed roles for *Leishmania* chitinase, the functional analysis of this enzyme in the vector has not been directly addressed to date. Recently, the *Leishmania mexicana* chitinase gene, *LmexCht1*, was identified and a homologous episomal expression system has been developed to express an epitope-tagged *LmexCht1* chimeric construct in these parasites (Joshi *et al*., 2005). These *Leishmania* were previously shown to overexpress chitinase in both amastigotes and promastigotes, which enhanced their intramacrophage survival and cutaneous pathology in mice. In this study we have utilized these parasites to evaluate the role of *Leishmania* chitinase in the parasite–fly relationship, its contribution to a successful sand fly infection and transmission to the mammalian host.

## Results

### Anatomy of *Leishmania*-infected sand flies

The anatomy of the midgut structures involved in bloodfeeding and *Leishmania* transmission are illustrated in Fig. 1. A cross-section through the head of an uninfected, female *Lu. longipalpis* sand fly (Fig. 1A) shows the mushroom shape of the SV in its normal closed position. The SV separates the proboscis and foregut from the thoracic midgut and lies dorsal to the paired salivary glands. Figure 1B is a diagrammatic representation of a cross-section through an infected sand fly, illustrating the position that *Leishmania* promastigotes and their gel-like plug (termed promastigote secretory gel, PSG) typically occupy during a mature transmissible infection. Formation of the biological plug involves colonization of the chitinous surface of the SV by the attachment of parasites and secretion of PSG by parasites in the gut lumen. In mature infections the biological plug extends forward and can force open the SV allowing parasites access into the foregut. Figure 1C and D show cross-sections through such a mature *L. mexicana* infection in *Lu. longipalpis*, illustrating the densely packed nature of the parasites, both attached to the valve and free in the gut lumen, and the distended open position of the valve resulting from the infection.

### Excess chitinase increases early mortality of *L. mexicana* in sand flies

**Fig. 2:**
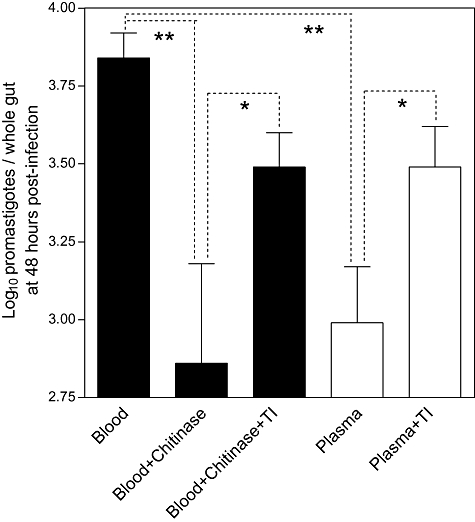
Excess chitinase in the blood meal exacerbates early mortality of *Leishmania* to sand fly midgut trypsins. Comparison of early growth of wild-type *L. mexicana* in blood or plasma supplemented with or without 1 mg ml^−1^ soybean trypsin inhibitor (TI) or 1 U ml^−1^ *S. griseus* chitinase. Data from a typical infection; each bar representing the average of 10 flies per group ±1 standard error from the mean. Asterisks indicate values that are statistically significant (*P < 0.05, **P < 0.01) using an unpaired *t*-test.

Previously, it was suggested that expression of chitinase by *L. major* in the sand fly *P. papatasi* during their initial transformation to promastigotes may actually be detrimental to survival by making the PM more porous and increasing exposure to trypsin-like enzymes (Pimenta *et al*., 1997). To investigate this possibility in our infection model, *Lu. longipalpis* sand flies were infected with wild-type (WT) *L. mexicana* amastigotes in blood supplemented with exogenous *Streptomyces griseus* chitinase at levels that were not harmful to *L. mexicana* growth *in vitro* (data not shown) and sampled at 48 h post infection before defecation of the digested blood and parasites (Rogers *et al*., 2002) (Fig. 2). Survival of parasites was severely compromised in these flies, showing a 90% reduction compared with controls (*P* < 0.05). However, inclusion of soybean trypsin inhibitor was able to restore infections toward control levels (55% of blood only control) and significantly higher than in the chitinase-only group (Blood + chitinase + trypsin inhibitor versus blood + chitinase = 422% increase in survival, *P* < 0.05). An alternative way of testing this hypothesis is to infect sand flies with amastigotes in plasma rather than whole blood, as this will also result in increased exposure to trypsin-like enzymes. Formation of the PM is unaffected but the lack of blood cell components increases the diffusion rate of these enzymes into the blood meal (Pimenta *et al*., 1997). Consistent with this, infections in plasma were significantly reduced compared with the whole-blood control (86% reduction, *P* < 0.05), but again was restored by the addition of soybean trypsin inhibitor (Plasma versus plasma + trypsin inhibitor = 316% increase in survival, *P* < 0.05).

### Overexpression of chitinase by *L. mexicana* facilitates survival and growth in sand flies

**Fig. 3:**
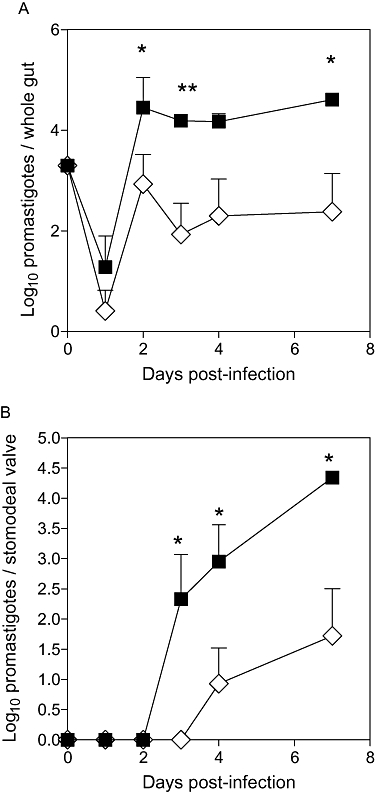
*Leishmania* chitinase assists in the infection of *Lutzomyia longipalpis* sand flies and colonization of the stomodeal valve. Flies were infected with plasmid control (*pKSNEO*; ◊) or chitinase-overexpressing (*pKSNEO::LmexCht1-HA*; ▪) *Leishmania mexicana* amastigotes. A. Kinetics of parasite growth in entire fly gut. B. The kinetics of parasite colonization and growth at the stomodeal valve region. Data from a typical infection; each point representing the average of 10 flies per group ±1 standard error from the mean. Asterisks indicate values from overexpressor infections that are statistically significant (*P < 0.05) from plasmid control infections using an unpaired *t*-test.

The results described above suggest that in sand flies early expression of high chitinase levels by *Leishmania* (1*–* 2 days post infection) would be potentially detrimental to survival (Pimenta *et al*., 1997), whereas previous work speculates that later expression (3–4 days post infection) would be beneficial (Schlein *et al*., 1992). To specifically observe the effect of parasite chitinase expression in sand flies, *Lu. longipalpis* were infected with chitinase overexpressor (*pKSNEO::LmexCht1-HA*) and plasmid control (*pKSNEO*) *L. mexicana* axenic amastigotes. The infections were sampled daily (Fig. 3A), and this revealed that during the first 24 h of development all infections suffered significant mortality (98–99% reduction). Interestingly, there was no significant difference between the two groups at the 24 h time point. Following this, both parasite lines recovered and infections with the chitinase-overexpressing parasites were consistently greater in number (*P* < 0.01) than the plasmid control parasites (and WT *L. mexicana* infections, data not shown) from 2 days post infection until the end of the experiment. Interestingly, on the third day of infection control parasites suffered a second large reduction in numbers (98–99%) from the previous day of infection. This coincides with the period during WT *L. mexicana* infection of *Lu. longipalpis* when the digested blood meal and PM are defecated from the fly (Rogers *et al*., 2002). By comparison, the chitinase overexpressor parasites could resist this second wave of parasite loss from the sand fly with only an average 42% of parasites lost or killed during this time.

### Overexpression of chitinase by *L. mexicana* facilitates escape from the peritrophic matrix and colonization of the stomodeal valve

After their escape from the digested blood meal and PM, *L. mexicana* promastigotes migrate anteriorly towards the SV where they resume cell division and complete the process of metacyclogenesis (Kamhawi, 2006). This anterior migration and avoidance of expulsion is assisted by their ability to attach to the midgut microvilli. To examine the effect of chitinase on this anterior migration a further set of infections were performed and the subpopulation of promastigotes present at the SV determined on a daily basis (Fig. 3B). Infections with plasmid control parasites (*pKSNEO*) were first detected on the fourth day post infection, in agreement with previous work using WT *L. mexicana* (Rogers *et al*., 2002). However, in the current study these were preceded by the chitinase overexpressing parasites (*pKSNEO::LmexCht1-HA*), which were detected on the third day of infection, 24 h ahead of plasmid control parasite infections. Throughout the infection the chitinase overexpressor parasites generated the highest valve burdens. By the end of the experiment (7 days post infection) the average valve burdens [±1 standard error (SE)] from infected sand flies were: *pKSNEO::LmexCht1-HA*: 2.24 × 10^4^ (±5.48 × 10^3^) versus *pKSNEO*: 68 (±2.64 × 10^2^), representing 58% and 27% of the total infections in the sand flies respectively.

### Overexpression of chitinase by *L. mexicana* increases the damage to sand fly stomodeal valve

**Fig. 4:**
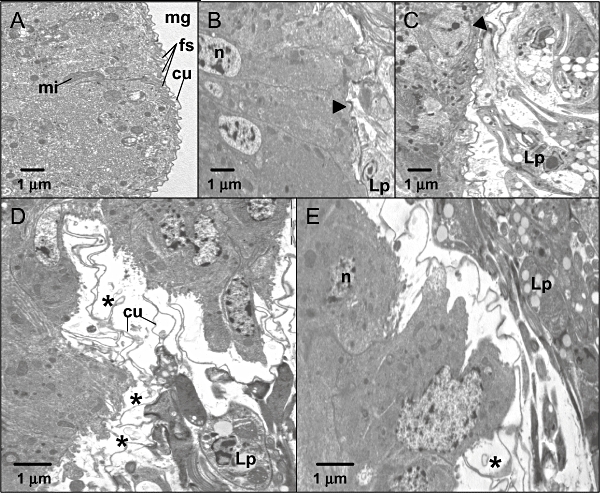
Expression of *Leishmania* chitinase in sand flies causes pathology to the stomodeal valve. Electron micrographs of stomodeal valves from: (A) uninfected sand flies, (B) wild-type, (C) plasmid control and (D and E) chitinase-overexpressing *L. mexicana*- infected sand flies. The position of filamentous structures in the stomodeal valve connecting the cylindrical epithelial cells of the valve to the chitinous cuticle is shown by lines. cu, cuticle; fs, filametous structures; mi, mitochondria; mg, midgut; n, nucleus of epithelial cells of the stomodeal valve; Lp, *Leishmania* promastigote. The arrowheads indicate hemidesome-like plaques on parasite flagellum and the asterisks indicate chitin fragments.

Previous work has suggested that *Leishmania* chitinase contributes to damage of the SV. To test this hypothesis, we compared the integrity of the SV in *Lu. longipalpis* following *pKSNEO::LmexCht1-HA* or *pKSNEO L. mexicana* infections using transmission EM (Fig. 4A–D). In uninfected flies (Fig. 4A) the columnar cells of the valve were intact and their apical ends in close association with the chitinous cuticle that forms a continuous and electron-dense layer on the outer surface of these cells. The columnar cells are attached to the chitin layer by the projection of short filamentous structures first observed by Volf *et al*. (2004). These structures are believed to play a contractile role and participate in the function of the SV. In flies infected with WT and the *pKSNEO*–transfected (plasmid vector control) *L. mexicana* the SV showed evidence of damage (Fig. 4B and C). In these flies the chitin layer of the valve was often separated from the apical end of the columnar cells. In some places there were fragments of electron-dense material between the chitin layer and the columnar cells which appears to be chitin. The filamentous structures in these infections appeared either stretched and/or absent. Despite these pathologies, in all control *L. mexicana* infections most columnar cells were intact with normal nuclei and mitochondria. The SV of flies infected with chitinase overexpressing *L. mexicana* displayed significantly more pathology (Fig. 4D and E). The columnar cells showed an absence of filamentous structures and a greater degree of separation from the chitin layer. The fragments of electron-dense debris were still visible in the gap between the cells and the chitin layer; however, they appeared more numerous. Compared with the control infections the columnar cells in the overexpressor infections appeared significantly eroded with an irregular shape and greatly reduced volume.

### Chitinase overexpression by *L. mexicana* in sand flies increases the time engaged in blood feeding

To test whether pathology to the SV could affect its functioning in blood intake, the time taken to blood feed was determined in flies with mature WT, *pKSNEO* control transfectant and *pKSNEO::LmexCht1-HA L. mexicana* infections. Flies were allowed to make only one blood feeding attempt on an anaesthetized mouse and the duration of feeding, size of fly infection and blood meal sizes recorded (Table 1). Previously, we have shown that WT *Leishmania*-infected *Lu. longipalpis* sand flies take longer to feed than uninfected sand flies (Rogers *et al*., 2002; Rogers and Bates, 2007). In this study flies infected with *pKSNEO::LmexCht1-HA L. mexicana* that overexpress chitinase spent on average 2.5 and 2.4 times longer to blood feed compared with both the WT and *pKSNEO L. mexicana*-infected flies, respectively, and 3.4 times longer than uninfected flies.

**Table 1:** Feeding activity of *Lu. longipalpis* sand flies infected with WT, *pKSNEO* and *pKSNEO::LmexCht1-HA L. mexicana* parasites.

Infection	Number of flies assayed	Average infection intensity (promastigotes/fly)	Sizes of blood meals (% **N**one, **P**artial or **F**ull blood meals)	Average duration of first blood feed (mean ± SE, s)
Uninfected	20	N/A	N: 0%, P: 0%, F: 100%	346 ± 45
Wild type	15	9.12 × 10^3^	N: 0%, P: 60%, F: 40%	471 ± 147
*pKSNEO* (plasmid control)	12	2.42 × 10^3^	N: 0%, P: 33%, F: 67%	487 ± 142
*pKSNEO::LmexCht1-HA* (chitinase overexpressor)	15	4.11 × 10^4^	N: 0%, P: 53%, F: 47%	1191 ± 352

N/A, not applicable.

### Chitinase overexpression by *L. mexicana* increases transmission to mice by sand fly bite

**Fig. 5:**
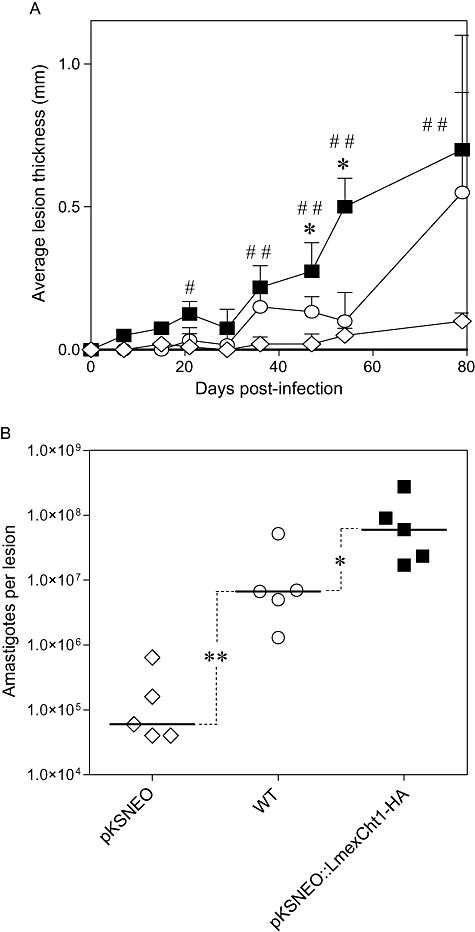
*Leishmania* chitinase overexpression enhances transmission from *Lutzomyia longipalpis* sand flies. Flies were infected with wild type (○), plasmid control (*pKSNEO*; ◊) or chitinase-overexpressing (*pKSNEO::LmexCht1-HA*; ▪) *Leishmania mexicana* amastigotes. Individual BALB/c mice were infected by single fly bites to the foot. A. Lesion development was monitored by measuring the thickness of the bitten foot compared with the unbitten foot. Each point represents the average lesion growth of at least five mice per group ±1 standard error from the mean. Asterisk and hash symbols indicate values from overexpressor infections that are statistically significant (*P < 0.05, #*P* < 0.05, ##*P* < 0.005) using an unpaired *t*-test from wild-type or plasmid control infections respectively. B. Final lesion parasite burdens from single fly bite infections of BALB/c mice. Bars represent the median value for each group and asterisk symbols indicate statistical significance between groups indicated (*P < 0.05, **P < 0.01) using the Mann–Whitney *U*-test.

The data so far have tested and described a role for *Leishmania* chitinase in the colonization of the sand fly vector and the possible adaptation of the fly for efficient transmission. Overexpression of chitinase resulted in increased pathology to the fly's SV and altered of the fly's blood feeding behaviour. Both of these effects could contribute to an increase in parasite transmission, as hypothesized by Schlein *et al*. (1992). Therefore, to directly test whether the overexpression of chitinase of *Leishmania* in sand flies could facilitate the transmission to and infection of the mammalian host we observed the evolution of the lesions that arose from the single fly feeds on individual mice (i.e. one infected fly bite per mouse) described above (Fig. 5). The lesions generated from the bites of the plasmid control (*pKSNEO*) *L. mexicana*-infected flies resulted in very small lesions by day 80, which contained very low numbers of amastigotes (average ± SE 1.88 × 10^5^ ± 1.15 × 10^5^). In contrast, the control WT *L. mexicana* infections yielded faster evolving and larger lesions containing approximately 77-fold more parasites at the end of the experiment (average ± SE 1.44 × 10^7^ ± 9.46 × 10^6^). However, the largest and fastest evolving lesions were generated from the bites of chitinase-overexpressing (*pKSNEO::LmexCht1-HA*) *L. mexicana*-infected sand flies, resulting in significantly larger lesions containing approximately 6.5- and 500-fold more parasites (average ± SE 9.32 × 10^7^ ± 4.73 × 10^7^) compared with the fly bite lesions from the WT and the control plasmid *L. mexicana* parasites respectively. These data clearly show that overexpression of *Leishmania* chitinase in the sand fly vector results in enhanced transmission to mice.

## Discussion

Chitinases hydrolyse the 1,4-β-linkages within chitin, a homopolymer of *N*-acetylglucosamine. Chitin is the scaffold of many structures within arthropods, including the PM and SV of sand flies (Walters *et al*., 1993; Secundino *et al*., 2005), which are also potential barriers to *Leishmania* infection. Chitinase has therefore been proposed to assist in the survival and establishment of *Leishmania* parasites within their sand fly vectors (reviewed in Sacks and Kamhawi, 2001; Bates and Rogers, 2004; Kamhawi, 2006), and for other vector-borne parasitic diseases. This study analysed the role of *Leishmania* chitinase in certain key aspects of the *Leishmania*-sand fly relationship that contribute to a successful sand fly infection and transmission to the mammalian host. This study is the first to use a molecular approach to directly test the importance of *Leishmania* chitinase in their sand fly hosts.

To do this we used *L. mexicana* transfected episomally with *pKSNEO* containing a copy of the chitinase gene. This resulted in the overexpression and secretion of enzymatically active chitinase by these parasites (Joshi *et al*., 2005). By using *pKSNEO* and *pKSNEO::LmexCht1-HA L. mexicana* axenic amastigotes in their first *in vitro* passage from lesion amastigotes under drug pressure we were able to retain both the general virulence of the parasites and a high copy number and prevalence of the plasmid (Cuvillier *et al*., 2003). These parasites along with WT were used to infect the permissive vector *Lutzomyia longipalpis* (Volf and Myskova, 2007), an experimental model of infection, which can support the growth and full development of *L. mexicana* and transmit these infections efficiently to mice (Rogers *et al*., 2002; 2004; Gossage *et al*., 2003).

Transforming amastigotes within the early blood meal environment are vulnerable to attack from sand fly midgut trypsins (Pimenta *et al*., 1997). During this time experimental infections of *Leishmania* typically suffer large mortalities in the order of 90% (Pimenta *et al*., 1997; Rogers *et al*., 2002), reflecting the importance of this barrier to sand fly infection. Working with *L. major* and *P. papatasi*, Pimenta *et al*. (1997) found that the formation of an intact PM was critical to the survival of parasites by limiting the early contact between the parasites and trypsins during this window of susceptibility. When *S. griseus* chitinase was included with their infections they found that the early parasite mortality was exacerbated, corresponding to the lack of an effective PM barrier. These workers also found that infections in plasma suffered the same mortality because it promoted the contact of parasites with sand fly trypsins without affecting the integrity of the PM. Although the enzymatic properties of *Leishmania* and bacterial chitinase may differ (e.g. pH and temperature optima) our results with *L. mexicana* in *Lu. longipalpis* are in good agreement with these previous findings. We found that WT *L. mexicana* infections suffered the greatest mortality during the first 24 h of the infection, but this could be partially rescued by supplying a trypsin inhibitor. Infections could be rescued in this manner either in plasma meals, or in blood supplemented with chitinase. Taken together, these data indicate that the high expression of parasite chitinase in the first 24 h would be detrimental to the early phase of the infection. Interestingly, in the current study when chitinase overexpressors were used to infect sand flies, we did not see an increased early mortality compared with controls. One possible explanation for this could be due to down regulation of chitinase in transforming parasites under *in vivo* conditions. This hypothesis, however, remains to be experimentally verified. Other explanations may include an inhibitory effect of haemoglobin on the parasite chitinase in the blood meal (Schlein and Jacobson, 1994), or simply that not enough chitinase is produced *in vivo* to promote this effect.

Following the successful transformation of amastigotes into flagellated procyclic promastigote forms, *Leishmania* need to escape the PM-encased blood meal in order to resist expulsion during sand fly defecation. To confirm the absolute need for this parasite enzyme at this point during sand fly infection a chitinase-specific *Leishmania* null mutant would be required (Dessens *et al*., 2001). This is not currently available; however, by using a parasite line that overexpresses chitinase we have shown how this enzyme can benefit the parasite at multiple points during the sand fly–parasite interaction. We found that the overexpression of parasite chitinase in the fly resulted in higher numbers of promastigotes able to breach the PM earlier and enter the thoracic midgut. This result could not be explained by differences in transformation rate, which were similar between the parasite lines observed *in vitro* and in the flies. However, it should be appreciated that the PM is not exclusively made from chitin, and is a matrix of other chitin-binding proteins and peptidoglycans (Lehane, 1997). Therefore, we do not discount the possibility that other *Leishmania* enzymes may contribute to their escape from the blood meal, for example, leishmanolysin (gp63) (Hajmová*et al*., 2004), a protease that may act in synergy with the parasite chitinase. Furthermore, in addition to the parasite chitinase-assisted escape from the PM-encased blood meal demonstrated here, it is also conceivable that at least some *Leishmania* species rely upon the fly's chitinases (Ramalho-Ortigão *et al*., 2005) and/or a combination of both activities to degrade their PM. This would provide *Leishmania* a short ‘window of opportunity’ during which they could escape the digested blood meal before defecation. Although sand flies vary in their digestive physiology it has been observed that they appear to initiate the degradation of their PM from the anterior and posterior ends (Walters *et al.*, 1989; 1993). Previously, we have shown that in *Lu. longipalpis* sand flies *L. mexicana* nectomonad promastigotes congregate at the anterior pole of the PM-encased blood meal immediately prior to defecation, suggesting that these parasite stages are adapted to take advantage of this window of opportunity (Rogers *et al*., 2002).

Beyond the blood meal phase of parasite infection we found that the overexpression of *Leishmania* chitinase resulted in an exacerbation of pathology to the SV extending the observations of Schlein *et al*. (1992) and Volf *et al*. (2004). The facilitated escape of parasites from the PM-encased blood meal allowed the chitinase overexpressor parasites to migrate to and colonize the anterior midgut and SV earlier than the other infections. Thus, larger infections were established at this important structure earlier, which may also contribute to the increased valve damage associated with these infections. Similarly, early blood meal escape was seen to benefit some WT *L. major* strains in *P. papatasi* (Cihakova and Volf, 1997). In mature *Leishmania* infections, including *L. mexicana*, the parasites block the anterior midgut by secreting a proteophosphoglycan-rich gel (Stierhof *et al*., 1999; Rogers *et al*., 2002). This is likely to further disrupt the functioning of the valve by forcing it permanently open (Jefferies *et al*., 1986; Rogers *et al*., 2002). Schlein and coworkers also hypothesized that a damaged SV may facilitate regurgitative transmission by allowing a reflux of blood and parasites back into the skin of the host during a blood feed. Therefore, to test whether the pathology resulting from *Leishmania* chitinase in mature infections affected the blood feeding ability of the flies and transmission to the mammalian host, we exposed flies infected with the control (WT and *pKSNEO* control transfectants) and chitinase-overexpressing parasites individually to mice. We found that flies with chitinase overexpressing parasite infections, and therefore more valve damage, experienced difficulty in feeding and took on average 2.5 times longer to feed. Significantly, this resulted in the generation of faster evolving cutaneous infections which lead to much larger lesions containing more parasites at the end of the experiment. This demonstrates that the parasite's chitinase is an important virulence factor leading to the deposition of more parasites into the skin of the host during blood feeding.

In conclusion, this study identifies the *Leishmania* chitinase as a multifunctional virulence factor that benefits the parasite throughout its entire life cycle. Building on our previous work (Joshi *et al*., 2005) that identified *Leishmania* chitinase to enhance the infection of the mammalian host, we show here that this molecule is also important for the parasite–vector interaction. In the sand fly we show that chitinase facilitates the escape of *L. mexicana* promastigotes from the PM-encased blood meal. This resulted in an earlier migration of promastigotes to the valve, thus, establishing the infection in a position suited for transmission. The *Leishmania* chitinase also contributed to SV pathology, as previously suggested (Schlein *et al*., 1992; Volf *et al*., 2004), and as a consequence of this pathology the contact time between the vector and the mammalian host was increased by reducing the rate at which the sand fly could blood feed. Finally we show that transmission of *Leishmania* to mice by sand fly bite was enhanced when the parasites overexpressed chitinase in their sand fly vectors. Taken collectively, our results demonstrate that the *Leishmania* chitinase can affect the environment in the sand fly vector to enhance parasite transmission.

## Experimental procedures

### Parasites

*Leishmania mexicana* amastigotes were isolated from infected BALB/c mice using the method of Bates (1994a,b) and allowed to transform at 26°C *in vitro* in promastigote medium (Medium199, 10%FCS, pH 7.2) at 5 × 10^5^ cells ml^−1^. Log phase *L. mexicana* promastigotes (in their first *in vitro* passage after transformation) were transfected with either the *pKSNEO* (control) or *pKSNEO::LmexCht1-HA* (chitinase overexpressor) plasmids (Joshi *et al*., 2005) by electroporation according to Debrabant *et al*. (2000). The transfectants were subsequently selected for their growth in increasing concentrations (up to 200 μg ml^−1^) of G418 in promastigote medium (initiated at 5 × 10^5^ cells ml^−1^) over a period of several weeks. To generate lesion amastigotes of these cell lines susceptible BALB/c mice (Charles River UK) were infected subcutaneously in the rump with 10^7^ late-stationary phase promastigote parasites. The promastigote growth and transformation kinetics of both *pKSNEO* control and *pKSNEO::LmexCht1-HA* transfected parasites were monitored following passage of lesion amastigotes into promastigote medium at 5 × 10^5^ cells ml^−1^.

### Sand fly infection

Sand fly infections were performed, maintained and sampled according to Rogers *et al*. (2002), with modifications. All infections were made with lesion amastigotes passaged once in axenic amastigote culture medium (Medium199, 20% FCS, pH 5.5) with (for *pKSNEO* and *pKSNEO::LmexCht1-HA* parasites) or without (for WT parasites) 50 μg ml^−1^ G418 (Invitrogen). Just prior to infection all parasites were washed three times in 1× phosphate-buffered saline (PBS), pH 7.2 (Invitrogen) and resuspened at a density of 2 × 10^6^ ml^−1^ in fresh rabbit blood. In a number of infections to assay the effect of a lack of trypsin or an excess of chitinase on parasite growth, sand flies were infected with WT *L. mexicana* in rabbit blood or plasma supplemented with 1 mg ml^−1^ soybean trypsin inhibitor (Sigma) or 1 U ml^−1^ *Streptomyces griseus* chitinase (Sigma), or both. After infection flies were sampled to assess the growth of the various infections. Numbers of parasites were determined by homogenizing the whole midgut or SV region in 30 μl 1× PBS, pH 7.2 using a Teflon-coated pestle in a 1.5 ml microfuge tube, and counted in a Neubauer haemocytometer. The developmental status of the parasites was determined by morphometric analysis of Geimsa stained smears according to Rogers *et al*. (2002).

### Microscopy of sand flies

Light and electron microscopy and was performed on infected flies day 7 post infection according to Volf *et al*. (2004). Briefly, whole flies were knocked down on ice and one-third of the abdomen was dissected away to allow efficient perfusion of the fly with ice-cold fixative (4% glutaraldehyde (Sigma) in 1× PBS, pH 7.2). After fixation at 4°C for 24 h the flies were washed extensively in 1× PBS and post-fixed in 1% osmium tetroxide for 1 h. Flies were then dehydrated in a graded ethanol series and propylene oxide and embedded into Epon. For light microscopy, semithin sections (1 μm thick) of WT *L. mexicana*-infected flies were stained with 0.01% toluidine blue and mounted with DPX. For light microscopy, photographs were taken with an Olympus BX51 microscope with a camera attachment. Ultrathin sections of the SV region were mounted on carbon-coated copper grids with Formwar film and stained with uranyl acetate and lead citrate and observed with 1200 JEOL electron microscope. Uninfected, blood fed females were processed in the same way and used as controls.

### Feeding assay and single fly bite transmission

Sand flies with 7-day-old WT, *pKSNEO* and *pKSNEO::LmexCht1-HA L. mexicana* infections and uninfected flies were exposed individually to an anaesthetized BALB/c mouse. The body of the mouse was screened to limit the site of feeding to the hind feet. The period of continuous feeding from the first insertion of their mouthparts until they were retracted was recorded. Following feeding each fly was knocked down on ice, dissected and the presence of an infection and the size of the blood meal (none, partial or full) recorded. The average infection level for the different groups of flies was determined from a sample of 10 flies from the whole population immediately before the feeding assay. The lesion development on these mice was monitored by measuring the swelling of the foot with Vernier calipers compared with the opposite, uninfected foot. At the end of experiments mice were humanely killed, their feet removed and parasite burdens determined by homogenization and counting. All procedures involving animals were approved by a local Animal Welfare Committee and performed in accordance with UK Government (Home Office) and EC regulations.

## References

[b1] Bates PA (1994a). Complete developmental cycle of *Leishmania mexicana* in axenic culture. Parasitol.

[b2] Bates PA (1994b). The developmental biology of *Leishmania* promastigotes. Exp Parasitol.

[b3] Bates PA, Rogers ME (2004). New insights into the developmental biology and transmission mechanisms of *Leishmania*. Curr Mol Med.

[b4] Cihakova J, Volf P (1997). Development of different *Leishmania major* strains in the vector sandflies *Phlebotomus papatasi* and *P. duboscqi*. Ann Trop Med Parasitol.

[b5] Cuvillier A, Miranda JC, Ambit A, Barral A, Merlin G (2003). Abortive infection of *Lutzomyia longipalpis* insect vectors by aflagellated *Ld* ARL-3A–Q70L overexpressing *Leishmania amazonensis* parasites. Cell Microbiol.

[b6] Debrabant A, Ghedin E, Dwyer DM (2000). Dissection of the functional domains of the *Leishmania* surface membrane 3′-nucleotidase/nuclease, a unique member of the class I nuclease family. J Biol Chem.

[b7] Dessens JT, Mendoza J, Claudianos C, Vinetz JM, Khater E, Hassard S (2001). Knockout of the rodent malaria chitinase *PbCHT1* reduces infectivity to mosquitoes. Infec Immun.

[b8] Feng LC (1951). The role of the peritrophic membrane in *Leishmania* and trypanosome infection of sandflies. Peking Nat Hist Bull.

[b9] Gossage SM, Rogers ME, Bates PA (2003). Two separate growth phases during the development of *Leishmania* in sand flies: implications for understanding the life cycle. Int J Parasitol.

[b10] Hajmová M, Chang K-P, Kolli B, Volf P (2004). Down-regulation of gp63 in *Leishmania amazonensis* reduces its early development in *Lutzomyia longipalpis*. Microb Infect.

[b11] Jefferies D, Livesey JL, Molyneux DH (1986). Fluid mechanics of bloodmeal uptake by *Leishmania* infected sandflies. Acta Trop.

[b12] Joshi MB, Rogers ME, Shakarian AM, Yamage M, Al-Harthi SA, Bates PA, Dwyer DM (2005). Molecular characterization, expression, and in vivo analysis of *LmexCht1*: the chitinase of the human pathogen, *Leishmania mexicana*. J Biol Chem.

[b13] Kamhawi S (2006). Phlebotomine sand flies and *Leishmania* parasites: friends or foes?. Trends Parasitol.

[b14] Lehane MJ (1997). Peritrophic matrix structure and function. Ann Rev Entomol.

[b15] Molyneux DH, Killick-Kendrick R, Peters W (1987). Morphology, ultrastructure and life cycles. The Leishmaniases in Biology and Medicine.

[b16] Pimenta PF, Modi GB, Pereira ST, Shahabuddin M, Sacks DL (1997). A novel role for the peritrophic matrix in protecting *Leishmania* from the hydrolytic activities of the sand fly midgut. Parasitol.

[b17] Ramalho-Ortigão JM, Kamhawi S, Joshi MB, Reynoso D, Lawyer PG, Dwyer DM (2005). Characterization of a blood activated chitinolytic system in the midgut of the sand fly vectors *Lutzomyia longipalpis* and *Phlebotomus papatasi*. Insect Mol Biol.

[b18] Rogers ME, Bates PA (2007). *Leishmania* manipulation of sand fly feeding behavior results in enhanced transmission. Plos Path.

[b19] Rogers ME, Chance ML, Bates PA (2002). The role of promastigote secretory gel in the origin and transmission the infective stage of *Leishmania mexicana* by the sandfly *Lutzomyia longipalpis*. Parasitol.

[b20] Rogers ME, Ilg T, Nikolaev AV, Ferguson MAJ, Bates PA (2004). Transmission of cutaneous leishmaniasis by sand flies is enhanced by regurgitation of fPPG. Nature.

[b21] Sacks DL, Kamhawi S (2001). Molecular aspects of parasite–vector and vector–host interactions in leishmaniasis. Annu Rev Microbiol.

[b22] Sacks DL, Perkins PV (1984). Identification of an infective stage of *Leishmania* promastigotes. Science.

[b23] Schlein Y, Jacobson RL, Shlomai J (1991). Chitinase secreted by *Leishmania* functions in the sandfly vector. Proc R Soc Lond Series B.

[b24] Schlein Y, Jacobson RL (1994). Haemoglobin inhibits the development of infective promastigotes and chitinase secretion in *Leishmania major* cultures. Parasitol.

[b25] Schlein Y, Jacobson RL, Messer G (1992). *Leishmania* infections damage the feeding mechanism of the sandfly vector and implement parasite transmission by bite. Proc Natl Acad Sci USA.

[b26] Secundino NF, Eger-Mangrich I, Braga EM, Santoro MM, Pimenta PF (2005). *Lutzomyia longipalpis* peritrophic matrix: formation, structure, and chemical composition. J Med Ent.

[b27] Shakarian AM, Dwyer DM (1998). The *Ld Cht1* gene encodes the secretory chitinase of the human pathogen *Leishmania donovani*. Gene.

[b28] Shakarian AM, Dwyer DM (2000). Pathogenic *Leishmania* secrete antigenically related chitinases which are encoded by a highly conserved gene locus. Exp Parasitol.

[b29] Stierhof Y-D, Bates PA, Jacobson RL, Rogers ME, Schlein Y, Handman E, Ilg T (1999). Filamentous proteophosphoglycan secreted by *Leishmania* promastigotes forms gel-like three-dimensional networks that obstructs the digestive tract of infected sandfly vectors. Eur J Cell Biol.

[b30] Volf P, Hajmova M, Sadlova J, Votypka J (2004). Blocked stomodeal valve of the insect vector: similar mechanism of transmission in two trypanosomatid models. Int J Parasitol.

[b31] Volf P, Myskova J (2007). Sand flies and *Leishmania*: specific versus permissive vectors. Trends Parasitol.

[b32] Walters LL, Modi GB, Chaplin GL, Tesh RB (1989). Ultrastructural development of *Leishmania chagasi* in its vector, *Lutzomyia longipalpis* (Diptera: Psychodidae). Am J Trop Med Hyg.

[b33] Walters LL (1993). *Leishmania* differentiation in natural and unnatural sandfly hosts. J Eukaryot Microbiol.

[b34] Walters LL, Irons KP, Guzman H, Tesh RB (1993). Formation and composition of the peritrophic membrane in the sand fly, *Phlebotomus perniciosus* (Diptera: Psychodidae). J Med Entomol.

[b35] WHO/TDR (2004). http://www.who.int/tdr/diseases/leish/default.htm.

